# Similarities and differences in underlying beliefs of socio-cognitive factors related to diet and physical activity in lower-educated Dutch, Turkish, and Moroccan adults in the Netherlands: a focus group study

**DOI:** 10.1186/s12889-016-3480-4

**Published:** 2016-08-17

**Authors:** Kristina Romeike, Latifa Abidi, Lilian Lechner, Hein de Vries, Anke Oenema

**Affiliations:** 1 Department of Health Promotion, School of Public Health and Primary Care CAPHRI, Maastricht University, Maastricht, the Netherlands; 2 Faculty of Psychology and Educational Science, Open University of the Netherlands, Heerlen, the Netherlands

**Keywords:** Nutrition, Physical activity, Determinants, Culture, Low educated, Ethnicity, Qualitative, Focus group

## Abstract

**Background:**

Unhealthy eating patterns and a lack of physical activity (PA) are highly prevalent in most Western countries, especially among lower-educated people, including people of non-Western origin. The aim of this study was to investigate and compare the beliefs and barriers that underlie socio-cognitive and planning constructs related to healthy eating and PA among lower-educated Dutch, Turkish, and Moroccan adults.

**Methods:**

Focus group interviews were conducted with 90 Dutch, Turkish, and Moroccan lower-educated adults between March and August 2012. Five semi-structured group interviews were conducted with Dutch participants, five with Turkish participants, and four with Moroccan participants. Men and women were interviewed separately. The question route was based on the Theory of Planned Behavior and self-regulation theories. The theoretical method used for the qualitative data analysis was content analysis. The interviews were recorded, transcribed, and analyzed by applying the framework approach.

**Results:**

Some participants seemed to lack knowledge of healthy eating and PA, especially regarding the health consequences of an unhealthy lifestyle. Important attitude beliefs concerning healthy eating and PA were taste and health benefits. Participants suggested that social support can encourage the actual performance of healthy behavior. For instance, exercising with other people was perceived as being supportive. Perceived barriers to PA and cooking healthily were a lack of time and tiredness. These previously mentioned beliefs arose in all the ethnic groups. Differences were also found in beliefs between the ethnic groups, which were mainly related to religious and cultural issues. Turkish and Moroccan participants discussed, for example, that the Koran contains the recommendation to eat in moderation and to take care of one’s body. Furthermore, they reported that refusing food when offered is difficult, as it can be perceived as an insult. Finally, men and women usually cannot exercise in the same location, which was perceived as a barrier. These factors did not emerge in the Dutch groups.

**Conclusions:**

The same cognitive beliefs were discussed in all three ethnic groups. The importance of cultural and religious factors appeared to be the most significant difference between the Turkish/Moroccan groups and the Dutch groups. Accordingly, interventions for all three ethnic groups should focus on socio-cognitive beliefs, whereas interventions for Turkish and Moroccan populations can additionally take religious and cultural rules into account.

## Background

Unhealthy dietary habits and a lack of physical activity (PA) can cause serious health problems, such as cardiovascular diseases, type 2 diabetes, cancer, being overweight, and obesity [1–4]. These risk behaviors are highly prevalent in most Western countries, particularly among lower-educated people, increasing their risk of health problems [5–9]. Hence, it is necessary to promote a healthy diet and a sufficient level of PA among this high-risk group. One option to achieve this is by means of an intervention that motivates lower-educated people to induce behavioral change. To develop an effective intervention, insight is needed into the antecedents and specific beliefs related to healthy eating and PA among the members of this group. The lower-educated population of the Netherlands includes relatively large numbers of people from ethnic minority groups. Overall, 11.9 % of the population of the Netherlands is of non-Western origin. The Turkish and Moroccan groups are the two largest non-Western minority groups in the Netherlands; together, they account for 39 % of the non-Western population [10]. Most of the Turkish and Moroccan people in the Netherlands have a lower education level [11], and unhealthy dietary and PA patterns have been reported among them [12–15]. Therefore, we included Dutch, Turkish, and Moroccan participants in this study.

Previous studies have shown that constructs derived from socio-cognitive theories, such as the Theory of Planned Behavior (TPB), can explain to some extent the dietary and PA behaviors among different populations [16–21]. Furthermore, some of the differences between dietary intake and PA patterns between high- and low-educated populations can be explained by the socio-cognitive factors derived from these theories. Studies from Australia and the United States have suggested that knowledge of nutrition, social support, considering health when making food choices, taste preferences, and costs partly explain fruit or vegetable consumption among lower-educated women [22–24]. Another study confirmed that knowledge of nutrition may explain some of the dietary differences between adults with a high socioeconomic status (SES) and people with a low SES [25]. Furthermore, barriers such as taste and a lack of time were found to be related to fruit and vegetable consumption among lower-educated, multiethnic populations [26]. A study among Dutch adults showed that attitude partly mediates the relationship between educational level and vegetable consumption [27]. PA among lower-educated/low-SES populations has been shown to be associated with perceived control, self-efficacy, perceived outcome expectancies, attitude, social support, modeling, and intention [28–30]. Qualitative studies have indicated that, among other factors, a lack of social support, taste preference, a lack of time, perceived high costs of healthy food, and holding on to traditional food practices may be barriers to healthy eating for women with a low SES [31, 32]. Examples of the factors that may influence PA among people with a low SES are the perception of the accessibility and costs of athletic facilities, positive beliefs about PA, and social support [33].

Qualitative data have revealed that hospitality and the social environment play an important role in the eating and cooking habits of Turkish and Moroccan people [34, 35]. Another qualitative study pointed out that the woman’s role and time-consuming activities within the family (cooking and taking care of children) may impede PA among Turkish and Moroccan women. In addition, not being allowed to wear loose clothes and engage in sports in which men and women are mixed can be a barrier [36]. Finally, factors such as costs, time, place, and language seem to play a role in PA among Turkish and Moroccan women [36].

The overview of the current evidence indicates that socio-cognitive constructs are related to dietary and PA behavior in the target group [22, 23, 25, 31–36], but there is limited evidence with regard to the underlying beliefs concerning the socio-cognitive constructs. A detailed understanding of specific beliefs is necessary to develop appropriate interventions that can be tailored to the most important beliefs of specific target groups. It is further expected that there are differences in the underlying beliefs of the socio-cognitive constructs between the three ethnic groups investigated in this study. Previous research has indicated that specific beliefs can differ between ethnic groups [37, 38]. Therefore, the aim of this study was to gain insight into the specific beliefs that underlie the socio-cognitive constructs related to healthy eating and PA among lower-educated Dutch, Turkish, and Moroccan adults and to identify potential differences in these beliefs among these groups.

We developed a theoretical framework consisting of the TPB and planning elements from self-regulation theories to guide this qualitative study and future intervention development. The TPB states that a person’s attitude, subjective norm, and perceived behavioral control (PBC) determine his or her intention to perform a particular behavior. The intention, in turn, determines whether a health or risk behavior is carried out [39]. We chose the TPB because this theory has been widely used in dietary and PA behavior studies and can predict these behaviors reasonably well [16–21]. Furthermore, the TPB has been used successfully in studies among lower-educated and ethnic minority groups [40–43]. The constructs in this theory are likely to be culturally independent, even though the weight and underlying beliefs may differ across population groups [44]. One limitation of the TPB is that it does not address the volitional phase of the behavior change process in which the translation of a positive intention into a behavior has to occur, which may indicate why the explained variance for behavior is often lower than expected. Therefore, we added the elements of planning to our model. The importance of planning is derived from self-regulation theories, and planning is thought to overcome the intention–behavior gap [45–48]. Thus, we will study the underlying beliefs regarding attitude, subjective norm, PBC, and planning related to diet and PA.

## Method

### Focus group design and participants

The study was designed as a series of focus group interviews. The focus group approach was chosen because the group interaction could produce new beliefs and more in-depth discussion and responses than could be expected from individual interviews. The theoretical approach to the qualitative work was content analysis [49, 50], more specifically the framework approach [51], since our aim was to generate and analyze responses within the theoretical framework we used to explain the behavior. Fourteen focus group interviews were conducted between March and August 2012. Participants were included if they were of Dutch, Turkish, or Moroccan origin, were living in the Netherlands, were between 20 and 65 years old, and had a lower education level (i.e. lower vocational level or below). Being of Dutch origin was defined as being born in the Netherlands and having parents who were both born in the Netherlands. Being of Turkish or Moroccan origin was defined as having at least one parent who was born in Turkey or Morocco. This definition is in line with the standard definition of foreigners as formulated by Statistics Netherlands [52]. People were excluded if they had insufficient command of the Dutch language and if they had any psychological or physical health condition that may influence their eating or PA behavior (e.g. diabetes, depression).

The requirement for language was set because this study is part of a larger project aiming to develop a computer-tailored intervention for lower-educated Dutch, Turkish, and Moroccan women living in the Netherlands. In the first stage, this intervention will be developed in the Dutch language. If it is successful, translation into Turkish, Arabic, and Berber will be considered. A screening tool was used to assess potential participants’ eligibility for this study. Five Dutch, four Moroccan and five Turkish focus group interviews were conducted. The smallest group consisted of 3 and the largest of 14 people. The pre-determined number of focus group interviews was 12. Since this study included three different ethnic groups from both sexes, it was decided to conduct two interviews per subgroup, separated by sex and ethnicity. The literature suggests a rule of thumb indicating a minimum number of two focus groups per subgroup [53]. However, we proceeded by recruiting Dutch women because we had two interviews with only 5 participants. Further, we conducted three interviews among Turkish women because one group ended before the interview could be finished. As we pre-defined the amount of interviews, data saturation was not considered. Each participant participated in one interview; no repeat interviews were conducted.

### Participant recruitment

The participants were recruited in mosques (and through activities organized in the mosques), community centers (and associated cafés) in low-SES neighborhoods, social and sport associations, and women’s or mothers’ centers and foundations (e.g. Turkish or Islamic foundations). Potential participants were approached in person by members of the research team or through a representative of the organizations described above. Another recruitment method was the distribution of flyers and advertisements in places where the target population may notice them, such as dropping flyers through the letterboxes of houses in low-SES neighborhoods. Potential participants could contact the researchers and subscribe to the study. The researchers explained the inclusion criteria and used the screening tool to assess eligibility. Moreover, the snowball sampling method was used to recruit more participants via people who had already agreed to take part in the study.

### Procedure

The interviews were conducted in the locations where the participants were recruited and at times that suited the participants best in order to make participation as straightforward as possible. Having set the place, date, and time, the researchers made a reminder call on the day before the interview to make sure that the participants would not forget the appointment and to emphasize the importance of their presence. Two female researchers (KR and LA) were present as moderators at each interview. LA moderated the interviews with all the Turkish and Moroccan groups, while KR moderated the interviews with the Dutch groups. One of the moderators made field notes during the interviews. As LA has an Arabic background, this approach seemed appropriate, since the participants could relate to her cultural origin. The group sessions lasted between one hour and two and a half hours and were conducted in the Dutch language. The interviews were broader in scope than diet and PA only, but the other topics (among others, ‘indicators of ethnic identity’) are not part of this study report. The discussion concerning healthy eating and PA lasted on average about 32 and 20 min, respectively. With the participants’ permission, all the interviews were audio recorded. At the beginning of each interview, the participants were assured that all the information discussed in the group session would be treated confidentially and that audio records would only be available for the two moderators. The researchers had no prior relationship with the participants. The researchers introduced themselves before each interview and explained to the participants the goals and reasons for conducting this research. Furthermore, it was explained that the data would be used for publication. The researchers asked for the participants’ consent (verbally). One Turkish woman did not provide consent and therefore did not participate in the study. At the end of each interview, the participants filled out a short questionnaire including questions about demographics, educational level, and job situation. The participants were rewarded with a €20 gift voucher. Table 1 provides information about the interview settings and the constellation of the groups.

**Table 1:** Focus group interview locations, number of participants, mean age, and duration per group

Group	Location	Number of participants	Mean age in years	Duration of the interview (in minutes)^a^
Dutch women 1	Community center	5	56	118
Dutch women 2	Country dance club	5	62	56
Dutch women 3	Community center	3	54	57
Dutch men 1	Community center	5	58	109
Dutch men 2	Community center	6	44	82
Turkish women 1	Mosque	8	39	145
Turkish women 2	Mosque	8	40	89
Turkish women 3	Mosque	6	45	80
Turkish men 1	Community center	6	24	104
Turkish men 2	Mosque	6	48	83
Moroccan women 1	Community center	14	49	130
Moroccan women 2	Women’s health center	5	46	96
Moroccan men 1	Mosque	8	46	101
Moroccan men 2	Mosque	5	49	88

^a^Includes more topics than diet and PA, but only the discussions about diet and PA are the focus of this study report

### Question route

The interviews were carried out following a semi-structured question route (Table 2). This question route was developed according to the suggestions made by Krueger [54]: (a) the introduction of each participant, (b) an introductory question to initiate each topic, (c) a transition question bringing the discussion to the key point, (d) key questions, and (e) an ending question to ascertain that no information is missing. Steps (b) to (d) were followed for both topics (diet and PA). The question route was developed around the discussion of the constructs described in the TPB and self-regulation models (i.e. attitude, subjective norm, PBC, and planning). Additionally, the introductory question was specifically formulated to assess the participants’ comprehension of healthy eating and a sufficient amount of PA. This question may reveal beliefs and conceptions concerning healthy eating and PA that help the researchers to better understand the respondents’ main answers to the transition and key questions. In addition to the question route, a checklist was prepared to make sure that no important topics were left out (Table 2). The checklist was based on the TPB (attitude, subjective norm, and PBC) and self-regulation theories (planning) as well as previous studies (economic factors, cultural factors) and contained a list of concepts that were expected to emerge during the interviews. If a concept from the checklist did not arise during the discussion, the moderators gave cues that stimulated the participants to talk about that specific concept. The question route was first pilot tested among a group of colleagues who provided feedback for improvement. Then the questions were pilot tested with four Dutch members of the target group (three women and one man).

**Table 2:** Semi-structured question route and checklist

Introduction	Tell us your name and what you favorite dish is.
Introductory question for topic 1^a^	What is healthy eating for you?
Transition questions for topic 1	Is it important for you to eat healthily?
	Why yes/no?
Key questions for topic 1	What makes it easy for you to eat healthily?
	What makes it difficult for you to eat healthily?
	Do you think that you will succeed in eating more healthily? Why yes/no?
Checklist for topic 1	
Concepts from TPB	PBC: do the participants feel able to eat healthily?
	Attitude: what is the participants’ opinion about healthy eating?
	Subjective norm: do the participants’ family members hinder or facilitate healthy eating?
Concepts from self-regulation theories	Planning: do the participants plan their eating practices in any way?
Previous studies	Culture: which cultural factors influence participants’ eating behaviour? E.g.: traditional food practices, norms, hospitality, religion
	Money: does money play a role in healthy eating?
Additional questions	If participants mention cultural factors, how could they manage to deviate from traditional or religious norms, habits, and practices with regard to unhealthy eating?
	If there are participants who eat healthily, how do they do it? How do they overcome barriers, tradition, and norms?
Introductory question for topic 2^a^	What is a sufficient amount of exercise for you?
Transition questions for topic 2	Is it important for you to exercise?
	Why yes/no?
Key questions for topic 2	What makes it easy for you to exercise?
	What makes it difficult for you to exercise?
	Do you think that you will succeed in exercising more? Why yes/no?
Checklist for topic 2	
Concepts from TPB	PBC: do the participants feel able to engage in PA?
	Attitude: what is the participants’ opinion about PA?
	Subjective norm: do the participants’ family members hinder or facilitate PA?
Concepts from self-regulation theories	Planning: do the participants plan their PA practices in any way?
Previous studies	Culture: which cultural factors influence participants’ PA behaviour? E.g.: tradition, norms, hospitality, religion
	Money: does money play a role in engaging in PA?
Additional questions	If participants mention cultural factors, how could they manage to deviate from traditional or religious norms, habits, and practices with regard to PA?
	If there are participants who engage in PA, how do they do it? How do they overcome barriers, tradition, and norms?
Ending question	Is there something important we should have talked about but that did not come up?

^a^Topic 1 = diet, Topic 2 = physical activity

### Analysis

The interviews were typewritten into transcripts and analyzed by one researcher (KR) using NVivo 9. This analysis was verified by a second researcher (LA). The results were discussed until a consensus between the researchers was reached. The framework approach was used to analyze the data. This approach is useful because the analysis can be conducted in both inductive and deductive ways [51, 55]. On the one hand, the transcripts were analyzed to identify statements that matched the concept of the TPB or self-regulation theory (deductive). On the other hand, statements that did not match the theory were identified. These could consist of unexpected information that answers the research question (inductive). Our decision to search for statements that match the theory was based on the use of content analysis (framework approach), which was used to find specific beliefs and barriers that can be used to produce tailored advice. The TPB and self-regulation theory were used to build the coding tree, for which we first formulated the main themes according to the research questions. These main themes were facilitating factors for healthy eating, barriers to healthy eating, facilitating factors for PA, and barriers to PA. Under each sub-theme, codes were added according to the TPB and self-regulation concepts, such as attitude, PBC, subjective norm, and planning. Additionally, other codes were added for more specific aspects, such as *money*, *time*, *being used to it*, *hospitality and culture*, *religion*, and *knowledge*. For each of these codes, more specific codes were added. For the code *attitude*, for example, codes such as *vegetables taste good* or *exercising is pleasurable* were added. We used these codes from the coding tree to create themes, and we used these themes to structure the results section (what is healthy eating/PA, attitude beliefs towards (un)healthy eating/PA, social influences regarding (un)healthy eating/PA, and barriers and solutions regarding (un)healthy eating/PA). The citations were translated from Dutch into English by KR. The transcripts and results of the interviews were not returned to the participants for feedback.

## Results

A total of 36 men and 54 women, aged between 22 and 73 years (mean age 46.2 years, SD = 12.6), participated in the interviews. Most of the interviewees (97.8 %) had a lower vocational education, primary school education, or no education at all. Two participants turned out to have a higher education (one graduated at a college of higher education, the other at a university in Morocco). Of the sample members, 29 % had paid work, whereas 44 % were housewives/-husbands. The remaining 27 % were incapable of work, unemployed, retired, or volunteers. Table 3 provides an overview of the participants’ characteristics. The results will be presented for each behavior separately and will be described in relation to each theme derived from the analysis. The first theme concerns how participants defined healthy eating and PA. Then, the results will be described according to the concepts of the TPB and self-regulation theory. Tables 4 and 5 provide a summary of the main themes and sub-themes that were discussed for each behavior. Citations will be presented for noticeable or frequent findings, accompanied by the origin, sex, and age of the cited participant.

**Table 3:** Overview of participant characteristics

	Dutch	Turkish	Moroccan
	(*n* = 24)	(*n* = 34)	(*n* = 32)
Sex			
Male	11	12	13
Female	13	22	19
Mean age	54.2	28.9	47.9
(Age range)	(23–66)	(22–56)	(31–73)
Education			
None	2	-	14
Basic education	3	14	8
Lower education	19	20	8
Higher education	-	-	2
Employment status			
Paid work	10	11	5
Incapable of work	-	2	5
Unemployed/seeking	2	1	2
Retired	5	-	2
Housewife/-husband	7	16	17
Volunteer	-	4	-
Other	-	-	1

**Table 4:** Main themes, sub-themes, and specific beliefs regarding healthy eating

Main themes^a^	Sub-themes^b^	Ethnic group
What is healthy eating?	Healthy food products	All ethnic groups
	Lack of knowledge	Dutch + Turkish
	Religious rules	Turkish + Moroccans
	Misconceptions	Turkish + Moroccans
Attitude beliefs towards (un)healthy eating	Health benefits	All ethnic groups
	Taste	All ethnic groups
	Costs	All ethnic groups
	Preparing food	All ethnic groups
	Feeling about food	Turkish + Dutch
Perceived subjective norm, social support, and social pressure regarding (un)healthy eating	Social support and pressure by spouses and children	All ethnic groups
Barriers and solutions regarding (un)healthy eating	Time	All ethnic groups
	Work	All ethnic groups
	Creativity	All ethnic groups
	Strategies to overcome barriers	All ethnic groups
	Hospitality/culture/religion	Turkish + Moroccan

^a^These themes were defined based on the sub-themes. The concepts from the Theory of Planned Behavior were used to formulate the main themes

^b^These themes arose during the discussion

**Table 5:** Main themes, sub-themes, and specific beliefs regarding PA

Main themes^a^	Sub-themes^b^	Ethnic group
What is a sufficient amount of PA?	Number of days and amount of time	All ethnic groups
	Daily activities	Turkish + Moroccan
	Religion	Turkish + Moroccan
Attitude beliefs towards PA	Health benefits	All ethnic groups
	Costs	All ethnic groups
	Feeling about PA	Dutch + Turkish
	Pleasure	Dutch + Turkish
Perceived subjective norm, social support, social pressure regarding PA	Social support by friends and family	All ethnic groups
	Exercising in a group	All ethnic groups
	Exercising alone	Dutch + Turkish
	Culture	Turkish + Moroccan
Barriers and solutions regarding PA	Time	All ethnic groups
	Busy schedule	All ethnic groups
	Motivation	All ethnic groups
	Mood	All ethnic groups
	Fatigue	All ethnic groups
	Strategies to overcome barriers	All ethnic groups

*PA* physical activity

^a^These themes were defined based on the sub-themes. The concepts from the Theory of Planned Behavior were used to formulate the main themes

^b^These themes arose during the discussion

### Healthy eating

#### What is healthy eating?

When asking the participants what healthy eating encompasses for them, all agreed that fruit and vegetables are part of a healthy diet. A few Turkish women added that vegetables from one’s own garden are better than those from supermarkets. The Turkish participants, as well as some of the Dutch men, stated that eating in moderation is healthy, implying that one should not eat too much at once. In all the ethnic groups, some participants mentioned that consuming little fat is healthy and that using olive oil is the best choice. In almost all of the groups, the participants mentioned meat and fish as being healthy products, even though many stated that one should not eat too much meat. Additionally, chicken was mentioned frequently as a healthy meat choice. In the Turkish groups, the participants brought up yogurt as a healthy food item, while some said that homemade yogurt is the best. They argued that yogurt contains good bacteria for the intestines. One Dutch woman mentioned that light products are healthy, such as diet coke, because they do not contain sugar.

Products with light; drinking diet coke. […] That contains less sugar. (Dutch woman, 65)

According to the Turkish and Moroccan participants, male as well as female, Islam recommends eating healthy food. The participants explained that the Koran advocates avoiding specific food items, such as sugar and salt.

We also have sugar, it is also in the Koran; [it] is dangerous for people. […] Mohammed said that you should avoid three things: sugar, salt, and white flour. (Moroccan woman, 37)

The female participants seemed sure about the meaning of healthy eating; men were more insecure. A few Turkish and Moroccan men stated that they did not know what healthy eating really is. A Dutch man pointed out the confusing messages that one receives from the media about healthy and unhealthy food. Milk, for instance, was known to be healthy in the past; nowadays, it is suggested that milk is not beneficial after all. A Turkish man explained that the lack of knowledge may be a result of the Turkish culture. He pointed out that he did not learn what healthy eating is; he learned that it was normal to eat a lot of meat, fat, and bread.

[…] we have very little information on what healthy eating actually is. And we did not really learn it in our culture. We are really a culture in which we eat a lot of meat and oil […]. We have irregular eating times. (Turkish man, 24)

Furthermore, the participants brought up some misconceptions concerning healthy and unhealthy food. One Turkish man stated, for example, that red meat is important for the body. In one Moroccan group, the misconception arose that diabetes and cancer are not caused by unhealthy eating and physical inactivity. Something similar was stated by a man in the other Moroccan group, who thought that it is not food that makes people sick but chemical substances in the food. Furthermore, a few Turkish and Moroccan participants stated that the food nowadays is not healthy anymore, because chemical substances and hormones are added to it, because it is genetically manipulated, and because it is no longer organic.

But I don’t believe that you get sick from the food. Look, that simply comes from chemical substances that are injected into meat, for instance. They have to deliver more products, so they make many chemical products; so, that is not organic anymore. (Moroccan man, 61)

#### Attitude beliefs towards (un)healthy eating

The main beliefs that emerged in almost all of the groups concerned the health benefits of a healthy diet, the taste of food, and the perceived costs of healthy food. In all 14 groups, the participants reported that healthy eating is important because it is beneficial for one’s health and because it can prevent weight gain.

Moderator: Is it important for you to eat healthily, too?

Moroccan woman (43): Yes, for [a] healthy body; [to] not [get] sick.

Some Turkish and Moroccan men said that they do not think about healthy eating, as they just eat what is made at home.

I don’t especially make healthy [food], but standard what I cook at home, [or] my wife, I just eat it. (Moroccan man, 45)

Dutch participants brought up the good taste of *healthy* food items as a reason for eating healthily. They stated that *healthy* food items were delicious, whereas certain *unhealthy* items were disliked.

I think it tastes better. Vegetables taste better. (Dutch woman, 65)

I never go to a snack bar. […] No, I just don’t find it delicious. (Dutch man, 23)

The Turkish and Moroccan participants did not mention taste as a reason to eat healthy food. A few Turkish and Moroccan men pointed out that they eat what they like, without taking into account whether it is healthy or unhealthy. Hence, they actually do care about taste, but the taste is not a reason for them to eat *healthy* food. Furthermore, all three ethnic groups mentioned “good taste” as a reason to eat *unhealthy* food items.

Sometimes you find something delicious, but [it] is not healthy; [it is] fatty. But you still make it. It is not healthy, but still. (Moroccan woman, 37)

Most of the participants did not think that healthy food is expensive, as long as it is not organic. The Dutch participants stated that they consider prices but that they just buy what they like. Some would choose cheaper products over organic products, while others already buy organic products. A few Dutch women stated that they prefer to buy vegetables that are in season and on sale but that a high price would not prevent them from buying vegetables. The Turkish men did not think that money plays a role in healthy eating, whereas the Moroccan men thought that some healthy food items, such as fish, are expensive. Other Turkish and Moroccan participants said that they consider the price of food, especially because they have a whole family to feed. They further pointed out that organic products are expensive, but that health is more important than the price.

I don’t find healthy food expensive […] Organic vegetables: those are expensive. But if I make a plan for eating for the whole week […], that is cheap. But the problem is organic. (Moroccan man, 46)

Besides these three main themes, other beliefs emerged that were, however, only supported by a small amount of the participants. These beliefs were, for instance, that healthy eating makes one feel good, that vegetables are part of a proper meal, that peeling apples is annoying, and that ready-made meals are easy to prepare.

#### Perceived subjective norm, social support, and social pressure regarding (un)healthy eating

Social support to eat healthily and social pressure to eat unhealthily were mentioned in all ethnic groups. The social influence mainly derived from family members, such as children and spouses. The Dutch women mainly talked about eating healthily with their partner, which makes it easier to actually eat healthily. The male participants from all the ethnic groups stated that they eat healthily if their wife or mother cooks healthy food.

If my wife adds salad or something to it, then I also eat salad. (Dutch man, 64)

The influence of the participants’ wives, however, could also work conversely: some of the Turkish and Moroccan men pointed out that they eat a lot whenever their wife cooks delicious food.

The problem is that we have women who prepare delicious food. That is the problem. We always eat; we eat a lot because we have women who prepare delicious food. (Moroccan man, 46)

For women from all three ethnic groups, the family influence derived mostly from the children. If the children demand unhealthy meals, the mother usually provides them. If that is the case, some women find it difficult to resist eating those products. The Dutch women further pointed out that they find it hard not to buy and eat snacks, such as chips, because their children ask for them. One woman stated that buying snacks for her children automatically makes her eat them as well.

And if you only get it [snacks] for the children, you still eat it after all. (Dutch woman, 54)

Some women stated that the preferences of their children did not influence their own eating behavior. Usually, they would just cook several options for themselves and the children.

#### Barriers and solutions regarding (un)healthy eating

Besides the attitude beliefs described above, the practical barriers to healthy eating were discussed. The most frequently raised barrier in all the groups was a lack of time. Due to a lack of time, the participants find it difficult to prepare a healthy meal. Related to that, the male participants stated that the amount of working hours or shift work restricted their ability to make healthy food choices. Both the lack of time and the working hours led them to buy a quick, unhealthy bite from a snack bar or vending machine or prepare ready-made meals such as pizza or deep-fried food. Women from all the ethnic groups experienced a lack of creativity in cooking. They found it hard to come up with ideas to maintain variation in their meals. Some of the women stated that they cannot cook the same meals every day, so they start thinking about what they can cook the next day.

Sometimes you have time to cook healthily or prepare food. But sometimes you don’t have time for that. What can you do in an hour? You quickly grab something from the freezer or so. And that is not healthy eating. […] You work, your husband works. You maybe come home around 6 o’clock. Then you have to order pizza or you cook something; and then everything must be quick, quick. (Moroccan man, 45)

The Turkish and Moroccan participants pointed out that hospitality can make it difficult to eat healthily. Refusing food when it is offered on a visit is regarded as an insult to the host. The participants find it difficult to say “no” in a situation like that. On the other hand, the host is pressured to offer food, as hospitality is an important norm in the Turkish and Moroccan cultures. Hence, both the host and the guest feel obliged to each other when it comes to food.

[…] when we visit each other the table is usually full. And then it is not nice to say no. And then they’re really like, “Come on. Eat”; and you get dragged along with the chumminess. Then it is more difficult. (Turkish woman, 34)

To overcome these kinds of barriers, the participants discussed possible ways of increasing healthy eating. Strategies to make healthy eating easier arose from the discussions, such as preparing food in advance and cooking according to a healthy recipe. In the Dutch groups, but less so in the Turkish and Moroccan groups, the participants argued that eating healthily is something you “just do”, without thinking about it.

Moderator: What motivates you to eat healthily?

Participant: There is no motivation; you just do it! […] I don’t always think about it. (Dutch woman, 66)

Another way to manage to eat healthily is to plan in advance what to eat. Even though it was not stated often, planning was perceived as a good strategy by some participants.

On Monday, I think about what I have to cook tomorrow. (Moroccan woman, 42)

The Turkish and Moroccan participants explained that in some situations it is acceptable to refuse food. Indicating that one is full is one possibility to refuse an offer in a friendly way. Furthermore, it is possible to try only a few bites. Some of the participants emphasized that this is only possible with family, good acquaintances, or friends. In other situations, showing one’s respect for the host’s hard work by eating the food is more appropriate.

If you go somewhere spontaneously, then something will be prepared […]. But then you can just say, “Thanks for everything, but I’ve just eaten.” (Turkish man, 43)

Although some of the participants said that refusing food can be appropriate in some situations, the high level of importance and the persistency of cultural rules were established during the discussion.

[…] you can’t change the people. The people grew up like this. That is the generation. (Moroccan man, 46)

### Physical activity

#### What is a sufficient amount of PA?

In all the groups the participants agreed that it is important to be physically active every day. Most participants referred to moderate activities, such as walking and biking. A small number of the Turkish and Moroccan participants thought that one should even engage in sports every day. The amount of time that should be dedicated to PA varied from 20 min to 2 h for moderate PA and from 30 min to 2 h for more intensive activities (e.g. fitness, running, dancing). The Turkish and Moroccan participants mentioned that daily activities, such as working, household chores, taking care of children, and praying, also count as PA. Most of them, however, believed that these activities are not sufficient. Moreover, the Turkish and Moroccan participants explained that Islam has specific rules concerning PA; according to the participants, their prophet recommended walking for 40 min every day.

Our prophet also gave advice for Muslim people: 40 min of walking per day. Our prophet says that. It’s Sunnah [rules for Muslims as taught by the prophet Muhammad]. (Turkish woman, 31)

#### Attitude beliefs towards physical activity

The most important beliefs concerning PA that the participants raised during the interviews were related to the health benefits and costs of PA. PA was important to the participants to keep up good health and high spirits and to decrease stress. The participants in the Turkish and Dutch groups reported that PA increases one’s fitness and condition. A few Moroccan participants stated that exercising helps to decrease one’s cholesterol level and the blood sugar level among diabetics. One Moroccan man argued that exercise helps to exude toxicants. The Turkish women mentioned that exercising is good for one’s heart, intestines, stomach, and the whole body. The Turkish men were less specific about the health benefits of PA, because they did not find PA to be important. The Dutch women mainly stated that they engage in PA because they want to maintain their mobility and prevent stiffness. Besides, they want to be able to walk normally as they become older. The Dutch men thought that PA was healthy as well, even though they did not state many specific beliefs.

But if you are fit, if you actively exercise, then perhaps you have a higher condition and more endurance to keep up your work better and longer. (Turkish man, 22)

Some of the Turkish and Dutch participants said that PA is inexpensive if it includes walking and biking. However, exercising was perceived as expensive if it includes exercising in a sport center and paying fees. This negative attitude belief was expressed in all three of the ethnic groups. One Turkish woman, for instance, stated that swimming and fitness involve costs, whereas walking is free of charge. A Turkish man specified that €60 per month for exercising seems expensive. On the other hand, in another group of Turkish men, one man said that he would spend money on exercise if he had more time for it. The Moroccan participants reported that they would engage in exercise if it was free of charge or costs little; one woman stated that €10–12 would be a feasible amount. Unfortunately, she did not mention whether she would spend this amount per month or per week.

Sports […] in a sport center cost money. That is a problem. Sixty euros per month is expensive. (Turkish man, 63)

Other beliefs that were reported during the interviews by a few Dutch and Turkish participants were that PA is pleasurable and that it makes you feel better.

But if you do it [exercise] for at least half an hour […], you release stress and go into nature; that gives me a good feeling. (Turkish man, 24)

#### Perceived subjective norm, social support, and social pressure regarding physical activity

For many of the participants, arranging an exercise appointment with a friend, family member, or group of people was perceived as an effective strategy to engage in PA. Most of the Dutch, Turkish, and Moroccan women stated that they regularly meet up with friends or family to engage in PA together, which makes it easier for them to be active. The male participants hypothesized that exercising in a group may be easier, but only a few stated that they actually take part. In contrast, some Dutch and Turkish men, as well as Turkish women, argued that exercising alone is not pleasurable and can be boring.

I can’t do it [exercise] alone; alone is difficult. If it is within a group, it’s easier, together with the women. (Moroccan woman, 43)

Turkish and Moroccan women emphasized that exercising is not a problem in their culture and that their men have no negative influence on their exercising behavior. However, a norm in some Turkish and Moroccan populations is that men and women are not allowed to exercise together in the same location. For most of the Turkish and Moroccan participants, this was a reason for not going to a sport center.

I have to say, in the Moroccan culture, they don’t exercise a lot most of the time. I think if everyone engages in exercise, you have to separate everyone. Women separate and men separate. If men are separate, I can go swimming almost every day or do fitness. But if everyone is together, that’s not possible. That’s not possible. (Moroccan man, 41)

#### Barriers and solutions regarding physical activity

In all the Turkish groups and most of the Moroccan and Dutch groups, the participants felt that a lack of time and having a busy schedule are barriers to being physically active. Many or irregular working hours were especially mentioned as a barrier by the male participants. Being busy with the children, household and grocery shopping, and spending time with family are examples that were mentioned by women. Some of the women, however, also mentioned working hours as a barrier. When asking some of the participants if they had time on the weekend, they stated that they would rather spend the weekend relaxing than exercising.

Lack of time. That is an important factor as well. For instance, if you have too many appointments and little time to do sports; that is difficult too. (Moroccan man, 61)

In addition, motivation, mood, and tiredness seemed to play a role in the PA behavior of most of the participants. Being busy for the whole day made the participants tired, which resulted in the lack of a good mood to exercise. These barriers emerged in all three ethnic groups.

Time and mood. When you’re home, you’re worn out. If you then still have to run, bike, or do fitness, you think about it twice: should I go or should I not go? (Dutch man, 58)

According to many of the participants, the best way to be physically active is to take their bike or walk whenever they go somewhere, such as shopping for groceries or visiting someone. This was stated in all the ethnic groups. A few Dutch participants and one Turkish man said that they “just do it” and that they are used to it. Another strategy to engage in PA, according to the Turkish and Moroccan men, is to make a plan about when to exercise.

Yes, making a plan. Tomorrow I jog half an hour. The day after tomorrow I jog one and a half hours. Something like that. (Turkish man, 39)

## Discussion

The aim of this study was to gain insight into and compare the underlying beliefs of socio-cognitive factors related to healthy eating and PA among lower-educated adults with Dutch, Turkish, and Moroccan backgrounds.

### Healthy eating

A large number of our participants had an adequate idea about what healthy eating encompasses. Consuming fruit and vegetables and eating in moderation were frequently mentioned as healthy behaviors. Almost all of the participants asserted that they considered healthy eating important because of its health benefits, but they seemed to be unaware of what the impact of healthy food actually includes. This emerges from the statements of some Dutch and Turkish participants who expressed that they were insecure about what healthy eating actually is, which may point to a perceived lack of knowledge, possibly caused by ambivalent media messages and cultural influences. Furthermore, a striking finding was that some of the Turkish and Moroccan participants held the misconceptions that certain diseases (e.g. diabetes) are not caused by unhealthy eating, which has been indicated before among general populations [56]. Other misconceptions were that the food quality in the Netherlands is poor and that healthy food is equivalent to organic food.

Even though research among general populations shows that a lack of knowledge is not a strong barrier for healthy eating [57], the findings from this study indicate that knowledge about what healthy eating encompasses and about the health behavior link may be improved within our target population. Further research should investigate the actual and perceived knowledge of this target group concerning healthy eating and its relationship with diseases and whether a (perceived) lack of knowledge hinders them to eat healthily. In the meantime, it could be beneficial to focus on increasing knowledge about the health behavior link, how food safety is handled in the Netherlands, and about the differences between organic and conventionally grown foods when developing lifestyle interventions. Conventionally grown foods were actually perceived as inexpensive among the participants, whereas organic foods were seen as expensive. The participants did not consider the costs as a barrier for healthy eating, which is in line with research among general populations [57]. Pointing out that the inexpensive, conventionally grown foods are healthy as well may improve the target group’s attitude towards these foods.

The social influences on women mainly derived from the food preferences of their children and husbands, whereas men shifted their responsibility to their wives, as they just eat what their wives cook. Previous studies have already found a relationship between social influences and eating behavior among low-SES groups, ethnic minority groups, and general populations [37, 46, 58, 59]. Interventions can focus on increasing social support for women by providing advice on how to resist social pressure from family members. The men from the current target group could be encouraged to re-evaluate and take responsibility for their own eating behavior.

Self-regulation principles to improve healthy eating were only marginally discussed in the interviews; only a few participants mentioned planning as a strategy to initiate healthy eating. This stands in contrast to research showing that self-regulation techniques are important in healthy eating behavior [60]. This may in part be due to the focus of our study, but it may also indicate a lack of self-regulation skills in our target population. Therefore, this topic warrants further research. As planning has been shown to be an important predictor of eating behavior [61], it may be a strategy that can be used in interventions for this target population.

The general beliefs related to knowledge, attitude, barriers, and social influences were quite similar in the three ethnic groups; therefore, they can be addressed in all of the three groups. The differences were especially related to cultural and religious factors, which were mentioned by the Turkish and Moroccan participants. The most important factors were hospitality and rules in Islam. Refusing food when offered during a visit is seen as an insult, hindering the Turkish and Moroccan participants from eating less on social occasions. This finding is in line with previous research [34, 35]. Some Turkish and Moroccan participants mentioned ways to circumvent the hospitality issue (e.g. only eating small amounts on social occasions). These strategies may be taken into account in intervention development efforts.

A further difference between the ethnic groups was that taste was mentioned by the Dutch participants as a reason to eat healthy food. The good taste of healthy food was not mentioned in the Turkish and Moroccan groups. The importance of taste preferences in relation to eating behavior has been studied before in general populations, indicating that people’s taste is related to what they eat [62, 63]. Stimulating our target group to try and learn to enjoy the taste of healthy food may improve their attitude towards healthy food items.

### Physical activity

Our participants seemed to know well what is considered to be a sufficient amount of PA. Some Turkish and Moroccan participants, however, thought that engaging in vigorous PA every day is necessary, which is a misperception that may affect their motivation to engage in PA, since it would be hard to meet this perceived norm. Future research should investigate Turks’ and Moroccans’ definition of a sufficient amount of exercise and whether their definition affects their exercising behavior and beliefs concerning PA. Informing the target group about the Dutch norm may give them a better idea about how much PA is actually required and by means of which activities a sufficient level of PA can be achieved.

The participants’ attitude towards PA was generally positive, even though only a few specific beliefs were raised. It may be that some of the participants do not perceive the advantages or disadvantages of PA, because they usually did not elaborate on them. The Dutch participants, for instance, said that they “just do it” (engaging in PA) without thinking about the advantages or disadvantages. Moreover, some Turkish participants stated that they feel better when exercising, which points to a more affective belief rather than thinking about the advantages and disadvantages of PA. The most accentuated attitude beliefs were related to the health benefits of PA, which is in line with research among general populations [46] and which indicates that they may be among the most important reasons for our target group to engage in PA. However, some Moroccan participants pointed out that they did not believe that PA is related to diseases. Putting emphasis on the impact of physical (in)activity in interventions may increase the target population’s knowledge as well as stimulate them to elaborate on the advantages of PA, which may improve their attitude towards PA.

Our results show that social support and the lack of it are important factors for engaging or not engaging in PA, a finding that has been reported in previous research among general populations [46, 59], including research among ethnic minority groups [64]. In a previous study among a similar target group, PA is even described as a joint social activity rather than as an individual one [35]. An effective strategy to motivate our target group to be physically active may be to stimulate them to meet up with others to exercise. Furthermore, Turkish and Moroccan participants raised gender issues as a strong reason to avoid exercise in a gym, as men and women are not allowed to exercise together in the same location. This has already been suggested in a previous qualitative study [36] and was not mentioned in the Dutch groups. A strategy to enhance PA could therefore be to stimulate the members of the target group to create an environment that integrates privacy and social support.

Furthermore, we found that more practical barriers such as lack of time may be a problem for the target group. These results are in line with previous studies among low-SES populations [33, 36, 59] as well as general populations and other ethnic groups [65–67]. The perceived lack of time may also be related to the participants’ statement that they feel too tired because of their work. In fact, some participants stated that they are happy to have time to relax on the weekend. Therefore, intervention strategies for PA enhancement should focus on showing the target group how to make time for PA and how to integrate PA into their busy schedule. Our results point out that the beliefs related to PA are mostly similar in the three ethnic groups with the exception of minor differences, such as gender issues.

Unlike our participants, other populations have mentioned costs as a barrier to being physically active [65–67]. Costs may be a barrier among our specific target group, but other barriers seem to be more salient. Self-regulation strategies, such as goal setting and self-monitoring, have also been shown to play a role in PA behavior in other populations [46, 68]. As with healthy eating, the self-regulation strategies that came up in the discussions were less varied, as only a few participants mentioned planning as a strategy to improve PA.

### Limitations and strengths of the study

In one of the Moroccan male groups, two men had a higher educational level, which did not fit with the inclusion criteria. The effects on the results are minimal, as the comments of the two higher-educated participants did not differ from comments of the lower-educated participants. A further limitation is that the results were analyzed by only one researcher and verified by another researcher. Bias may be present in the interpretation of the interviews, and the results should be interpreted with caution. Moreover, we cannot draw conclusions about specific sub-behaviors of (un)healthy eating and physical (in)activity, since we only investigated the beliefs and barriers related to dietary and PA behavior in general. In this regard, the participants may have referred to different kinds of behavior during the interviews. Furthermore, the interviews were conducted in Dutch, which was problematic for some of the Turkish and Moroccan participants whose Dutch language skills were limited. It was sometimes necessary to provide examples to clarify the question.

We may have over-selected participants who already have an interest in healthy eating and PA. These participants may already have positive beliefs about healthy eating and PA. The attitude beliefs among our participants in particular were positive. Due to this potentially selective study group, we may have missed important negative attitude beliefs among the target group, which may be important to address in interventions.

Another limitation of the study is that, by focusing the study specifically on underlying beliefs for individual cognitions, we did not take the social or physical environmental context into account. This may, however, be a very influential context, especially for lower-educated people and people from more collectivistic cultures. Therefore, even though the approach taken was suitable for answering our research questions, it should be taken into account that environmental factors should also be addressed when developing comprehensive health-promoting interventions, especially for disadvantaged groups.

A strong point of this study is that the results add to the existing literature by pointing out the similarities and differences between the three ethnic groups. Other qualitative studies among this target group have mainly focused on social and cultural influences [34, 35] or only focused on ethnic minority groups without providing a comparison with the Dutch population [34, 36, 69].

## Conclusion

The Dutch, Turkish, and Moroccan participants generally stated similar beliefs and barriers concerning healthy eating and PA. These beliefs and barriers were mainly related to knowledge, attitude, social influences, and PBC. The main differences between the groups were that Turkish and Moroccan participants raised issues related to religion and culture, which were not discussed in the Dutch groups.

We conclude that the beliefs related to knowledge, attitude, social influences, and PBC are quite similar between the ethnic groups and that the differences lie in the cultural characteristics such as norms concerning hospitality, gender roles, and religion. These factors can be addressed in interventions for the Turkish and Moroccan target population. As for the beliefs related to knowledge, attitude, social influences, and PBC, we conclude that all beliefs can be addressed in all of the three ethnic groups. Based on our results, we cannot conclude that particular beliefs should or should not be addressed in one of the groups.
